# Hunter estimates of game density as a simple and efficient source of information for population monitoring: A comparison to targeted survey methods

**DOI:** 10.1371/journal.pone.0256580

**Published:** 2021-08-23

**Authors:** Jan Hušek, Melanie R. Boudreau, Marek Panek

**Affiliations:** 1 National Museum, Prague, Czech Republic; 2 Department of Biology, University of Hradec Králové, Hradec Králové, Czech Republic; 3 Department of Wildlife, Fisheries, & Agriculture, Mississippi State University, Mississippi State, Mississippi, United States of America; 4 Research Station, Polish Hunting Association, Czempiń, Poland; University of Bucharest, ROMANIA

## Abstract

Hunters in Europe gather non-survey game species population estimates to inform wildlife management, however, the quality of such estimates remains unclear. We compared estimates of game density, realized annual intrinsic growth rates, and period mean growth rates between hunter obtained data and data obtained by targeted survey methods for four species in Poland from 1960 to 2014. Raw hunter estimates were strongly positively correlated to spotlight counts of red fox (18 years of monitoring), strip counts of brown hare (21 years) and grey partridge (25 years), male call counts of partridge (24 years), and complete counts of roe deer (49 years), and not related to spotlight counts of brown hare (15 years). Realized annual intrinsic growth rates derived from hunter estimates were strongly positively related to annual intrinsic growth rates derived from strip counts of grey partridge and complete counts of roe deer, but only weakly or not related to strip counts of brown hare, spotlight counts of red fox and brown hare, and male call counts of grey partridge. The period length at which the period mean growth rates derived from hunter estimates and estimates from other methods were strongly correlated was largely variable among methods and species. In the roe deer, correlation between these variables was strong across all years, while in smaller game species the period mean growth rates based on hunter estimates and other methods had the strongest association in period lengths of 6 to 11 years. We conclude that raw hunter estimates convey largely similar information to that provided by other targeted survey methods. Hunter estimates provide a source of population data for both the retrospective and prospective analysis of game population development when more robust estimates are unavailable.

## Introduction

Hunting is a vital tool which can be used in the management of the game species populations [1–6]. In central Europe, hunters form hunting clubs lease a hunting territory and are responsible for sustaining viable populations of hunted game species, promoting habitat conservation, and buffering local communities against the effects of game species on agricultural practices [7–9]. Under such conditions, hunters play a role as a critical link between desired game species management, community agricultural practices, and habitat conservation [10], though understanding the interaction between hunter-based management and effects on game populations is still far from complete.

The way the hunting limits are set is often dependant on estimates of animal abundance and density, indicating that good population estimation is a critical part of sustainable game species population management [2,11,12]. Plenty of sound approaches, methods, and models have become available for estimation of animal abundance and density including distance sampling or capture-mark-recapture/resight; powerful tools allowing for modeling of detection probabilities and greatly improving the precision of estimates [13]. Yet, these methods have only rarely been adopted by hunting and game management authorities at the expense of much simpler approaches, such as hunter-based population estimates, presumably because of the inherited need for intensive expert-level manpower, high statistical competence for data analysis, and overall higher financial costs [14,15]. This may have fundamental consequences for game populations as it is rather uncommon that hunting authorities define census methods to be used by hunters for monitoring game abundance [7,16–20] nor supervise or coordinate their implementation, therefore, applied census methods may vary greatly between hunting areas [18].

Counting of game abundance by hunters prior to breeding season is a common practice in the monitoring of game population numbers in many countries with central European/Germanic hunting tradition (i.e., Poland, Czech Republic, Slovakia and other countries; [7,17,19,21]). Counts are performed over a few days to weeks prior to the breeding season at many sites evenly scattered throughout the hunting territory to derive mean game densities. After counts are performed, local hunting authorities set bag limits and define hunting plans based on hunter-collected estimates which are adjusted using estimates of reproductive output for each game species. Studies on large game generally suggest that the reported number of seen animals by hunters may reflect true densities, but the degree of precision varies greatly among species. For example, the number of moose (*Alces alces*) observed per hunter and hunting day at the beginning of a hunting season correlated only weakly or moderately with moose density [22–25]. In contrast, correlation between the number of seen red deer (*Cervus elaphus*) and population size was high in a study from Kvinnherad, Norway [26]. Estimates collected by hunters in a Finland yielded similar population trends in capercaillie (*Tetrao urogallus*), black grouse (*Tetrao tetrix*), hazel grouse (*Tetrastes bonasia*), and willow grouse (*Lagopus lagopus*) as estimates collected by ornithologists [16,27]. These, among many more examples across Europe [20,21,28], highlight how pervasive hunter estimates may be utilized for estimation of game densities.

One of the few emerging features from these comparisons is that hunter estimate precision typically decreases at higher population densities and increases with the openness of surveyed habitat [18,21,22]. In fact, the abundance of factors which can influence estimates leads to a prevailing skepticism in the utility of hunter estimates as robust measures of population size [18,26]. Additionally, while hunting clubs may be imposed with financial sanctions when failing to ensure legally binding duties, such as preventing crop damage by overpopulated game, there have been reported instances of hunting clubs providing distorted or even consciously manipulated abundance data to avoid such consequences [29]. On the other hand, good knowledge of the hunting territory and its game stands by local hunters may lead to improved precision of density estimates, even when some common-sense estimating of abundance may be involved. Surprisingly, little effort has been devoted to understanding the utility of hunter estimates beyond serving as a proxy for game densities (e.g., in a study of population dynamics; but see [20,22,24–26]). Clearly, there is an urgent need to validate hunter estimates of game abundance across taxa and time in countries which use estimates to set game population harvest levels against other reference methods.

We tested the relationship between hunter estimates and various targeted standardized survey methods across multiple species, including red fox (*Vulpes vulpes*), brown hare (*Lepus europaeus*), grey partridge (*Perdix perdix*), and roe deer (*Capreolus capreolus*), from a single hunting territory in western Poland using monitoring data spanning from 1960 to 2014. Our study aimed to evaluate how much of the same information about population density and development may be provided by hunter estimates and estimates from other survey methods under the same field conditions. Specifically, we aimed to test if i) hunter estimates of relative game density were positively correlated to density estimates derived by targeted survey methods, and ii) there was a positive correlation between realized intrinsic per capita annual growth rates and mean period growth rates derived from hunter estimates and estimates obtained by other targeted survey methods.

## Materials and methods

### Ethics statement

Our fieldwork was performed as a part of the long-term project "Monitoring game animals in Poland" conducted at the Research Station of the Polish Hunting Association in Czempiń, Poland (RS hereafter). This fieldwork was done in accordance with the valid legislation and no permit was required.

### Study area

Our study area (ca. 100 km^2^) is located in a hunting territory managed by RS near Czempiń, Poland (52°08’N, 16°45’E). The study area consists mostly of arable land with interspersed patches of mixed deciduous forests [30]. Roe deer, red foxes, brown hares, and grey partridge are common game species in Poland [7] and are hunted at the study area. While populations of roe deer and red fox increased from the late 1970s to 2000s across Europe, including Poland [31–34], those of brown hare and partridge have declined rapidly over the last 50 years [7,35,36]. Game management was conducted according to the central European/Germanic hunting tradition (i.e., via hunting territory management through a hunting club; [37,38]). One hunting season covers a period from 1^st^ April to 31^st^ March the following year. Further details on the regulation and organization of hunting in Poland has been provided by [7].

### Monitoring of game density

Minimal dataset is available in S1 Dataset.

### Hunter estimates

In late winter/early spring (February-March), as a by-product of conducting the main duties, hunters, gamekeepers, and managers from RS make notes on the number of seen individuals of brown hare, grey partridge and roe deer and, as a direct measure of a number of resident fox breeding pairs, the location of active fox dens (Table 1). Estimates of density in brown hare, grey partridge and red fox were derived by dividing the hunter field counts (i.e., the approximation of the population size) by total area of the hunting territory. Roe deer population density was derived by combining hunter estimates and estimates yielded from drive counts organized periodically until 1998 by foresters from State Forest Offices. State Forest Offices used drive counts in forests to estimate the average density of large game species in forests in the larger managed forest region, which includes our study area (in total 20 hunting districts with a total area of 108 000 ha, forested area 15 000 ha). Foresters selected random forest sections of size ca. 100 ha across the forest region (one section per 1000 ha). A group of observers surrounded each surveyed section at its borders and counted all big game flushed by a group of beaters who slowly walked through the surveyed section. Hunting managers in cooperation with foresters, use the late-winter/early-spring hunter estimates as a basis for setting maximum yearly bag size allowance for all four game species for the whole hunting district, considering the long-term population trends.

**Table 1:** Types and the period of surveys used to estimate relative game densities for red fox (*Vulpes vulpes*), brown hare (*Lepus europaeus*), grey partridge (*Perdix perdix*), and roe deer (*Capreolus capreolus*) at Czempiń, Poland from 1960 to 2014.

Species	Hunter estimate/targeted survey	Personnel	Period	Pros	Cons
Red fox	NSC of dens/ spotlight count	h, g, m/ sRS	1957–2014/ 1997–2014	Cost-effective, local knowledge within hunter community/suitable for larger game.	Unknown effort and precision, risk of adjusting to assuage political pressures, risk of missing dens/dubious relationship with true density, particularly so in small and elusive game species. Unclear suitability for comparisons between areas and years [39], the possible bias in sex and age ratios [40].
Brown hare	NSC/strip count	h, g, m/ sRS	1957–2014/ 1960–80, 84, 88	Cost-effective, local knowledge within hunter community/time and cost-effective, strong correlation with other census estimates even when values unadjusted for detection probability [41].	Unknown effort and precision, risk of double counting, and adjusting to assuage political pressures/need to account for detection probabilities. Census routes are chosen based on practical rather than theoretical reasons.
Brown hare	NSC/spotlight count	h, g, m/ sRS	1957–2014/ 1997–02, 06–14	Well established for monitoring hare.	Unknown effort and precision, risk of double counting, and adjusting to assuage political pressures/dubious relationship with true density, particularly so in small and elusive species. Unclear suitability for comparisons between areas and years [39], possible bias in sex and age ratios [40].
Grey partridge	NSC/strip count	h, g, m/ sRS	1957–2014/ 1966–1985	Cost-effective, local knowledge within hunter community/time, strong correlation with estimates from other survey techniques even when values unadjusted for detection probability [41].	Unknown effort and precision, risk of double counting, and adjusting to assuage political pressures/need to account for detection probabilities. Census routes are chosen based on practical rather than theoretical reasons.
Grey partridge	NSC/plot count using dogs	h, g, m/ sRS	1957–2014/ 1986–1990	Cost-effective, local knowledge within hunter community/well established in small galliformes [42,43]. Correlates with distance sampling estimates [44] and hunting bags [45].	Unknown effort and precision, risk of double counting, and adjusting to assuage political pressures/need for trained dogs. Possible stress for flushed birds. Lower efficiency than complete census and male call counts [44].
Grey partridge	NSC/male call count	h, g, m/ sRS	1957–2014/ 1991–2014	Cost-effective, local knowledge within hunter community/reasonable approximation of true density even with unadjusted values, also at low densities [41,46–48].	Unknown effort and precision, risk of double counting, and adjusting to assuage political pressures/manpower demanding. Possible nonlinearity in the relationship with true density [47].
Roe deer	NSC + drive count/complete counts	h, g, m, f/ h, sRS, f	1957–2014/ 1966–2014	Cost-effective, local knowledge within hunter community. Strongly related, or even superior, to distance sampling estimates [44,49].	Unknown effort and precision, risk of adjusting to assuage political pressures. Low accuracy at low densities and in spatially or demographically aggregating animals [50]/risk of double counting, manpower demanding.

Hunter estimates are a result of non-standardized counts (NSC) and specific targeted surveys. Personnel conducting surveys includes: f = foresters from the Forest State Offices, g = gamekeepers, h = hunters, m = hunting managers and, sRS = scientific staff of the Research Station. Potential pros and cons of survey methods are provided. Items specific to each estimation technique are separated by a forward slash.

#### Targeted survey methods

Targeted survey methods have been applied to monitor population numbers of game species as a part of various research projects of the RS.

*Strip counts*. Strip counts was used to estimate densities of brown hare (1960–1980, 1984 and 1988, Table 1) and grey partridge (1966–1985, Table 1). During days of non-inclement weather from late-February to early-April, a time during the year when count data is more accurate due to the lack of high crops which improves visibility for flushing hares [51]. A line of beaters (1 person/15 m) moved along a ca. 100 m wide strip covering various types of habitats. Several (n = 5–6) such strips were evenly distributed throughout the study area and had a total length of 57–75 km depending on the year of study. All hares and partridges flushed within the strip were counted. From 1986 to 1990 no beaters were used to flush partridges. Instead, partridges were located every March by a trained pointing dog on selected plots with a total area of 10.1 km^2^ (e.g., [43]). We derived hare density estimates by reducing the strip counts by 20% as it has been shown that the strip counts tend to be, on average, 20% higher than complete census counts in brown hare [51,52]. For partridge, strip counts underestimate complete counts at low partridge densities and previous work has corrected density using a linear relationship [52], however, we found that this yields unreasonable values at low densities, therefore, using data from [52], we derived a slightly modified nonlinear correction factor to be applied in our study, so that partridge density = 0.225 + 1.332*x* - 0.013*x*^2^, where *x* stands for strip count density (individuals km^-2^).

*Male call counts*. From 1991–2014, partridge abundance was estimated from mid-March to mid-April by counting calling males at 15 permanent monitoring sites distributed through the study area [47]. Mean number of calling males per monitoring site (*x*) was transformed to density as individuals km^-2^ = 3.38*x*^1.11^ [47].

*Spotlight counts*. While slowly driving a vehicle along a consistent route over the course of 3–5 nights from mid-March to early-April, observers counted all foxes and hares seen within a 200 m and 150 m wide strip, respectively, of illuminated land (Table 1). The route consisted of 10 sections, each being 2–7 km long (total length was 53 km until 2005, 33 km afterward). Foxes were counted from 1997 to 2014 [53], brown hares from 1997 to 2002 and from 2006 to 2014.

*Complete counts*. The grouping of roe deer in herds in fields from late autumn through spring allows for an accurate estimation of population abundance [54]. From 1966 to 2014, in late-February to early-March, on sunny and windless days, throughout the hunting territory, three or four observers used binoculars or telescopes to count all individual roe deer staying in open fields from a vehicle. Individual open agricultural fields not exceeding 100 ha allowed for increased detection of individuals. Observers made efforts to flush roe deer possibly hiding in any small woodlots to open places where they could count them. Additionally, during days of non-inclement weather from late March to early April, deer using larger forest patches for resting during the day were counted in the evening while feeding in the agricultural fields [30]. The numbers of roe deer flushed from small woodlots and counted during the day and those counted during feeding in the evening were summed up and considered as the complete counts (Table 1).

### Statistical analysis

We did not have any *a priori* indication of any causal relationship between hunter estimates and estimates derived by other targeted survey methods. Hence, we tested associations between variables by Pearson correlation. First, we analyzed relationships of hunter estimates of relative density to density estimates derived by other survey methods. Next, we tested the strength of association between realized intrinsic *annual* population growth rates (*r*_*t*_) obtained from hunter estimates and annual population growth rates estimated from other survey methods [22,26]. Realized intrinsic annual population growth rates were obtained as *r*_t_ *=* ln(density estimate)_t_—ln(density estimate)_t-1_.

Finally, we explored the strength of association between *period* mean population growth rates (${\bar{r}}_{\mathrm{period}}$) obtained from hunter estimates and period mean population growth rates obtained from other survey methods in relation to period length (i.e. number of years which the annual population growth rates were averaged over to obtain period mean population growth rates). Hence, for example, for the spotlight counts of red fox we obtained n = 16 mean growth rates for the period length of 2 years (${\bar{r}}_{1998–1999}$, ${\bar{r}}_{1999–2000}$, ${\bar{r}}_{2000–2001}$,… ${\bar{r}}_{2013–2014}$) but only n = 5 period mean growth rates for the period length of 13 years (${\bar{r}}_{1998–2010}$, ${\bar{r}}_{1999–2011}$, ${\bar{r}}_{2000–2012}$, ${\bar{r}}_{2001–2013}$, ${\bar{r}}_{2002–2014}$). Then, we estimated the strength of association to mean growth rates accordingly obtained from hunter estimates. In obtaining mean growth rates ${\bar{r}}_{\mathrm{period}}$ for the red fox spotlight counts, we considered period lengths of 2–13 years; for brown hare spotlight and strip counts periods 2–4 years and 2–16 years, respectively; for grey partridge strip and male call counts period lengths of 2–20 and 2–19 years, respectively; for roe deer complete counts periods of 2–44 years were considered.

## Results

**Fig 1:**
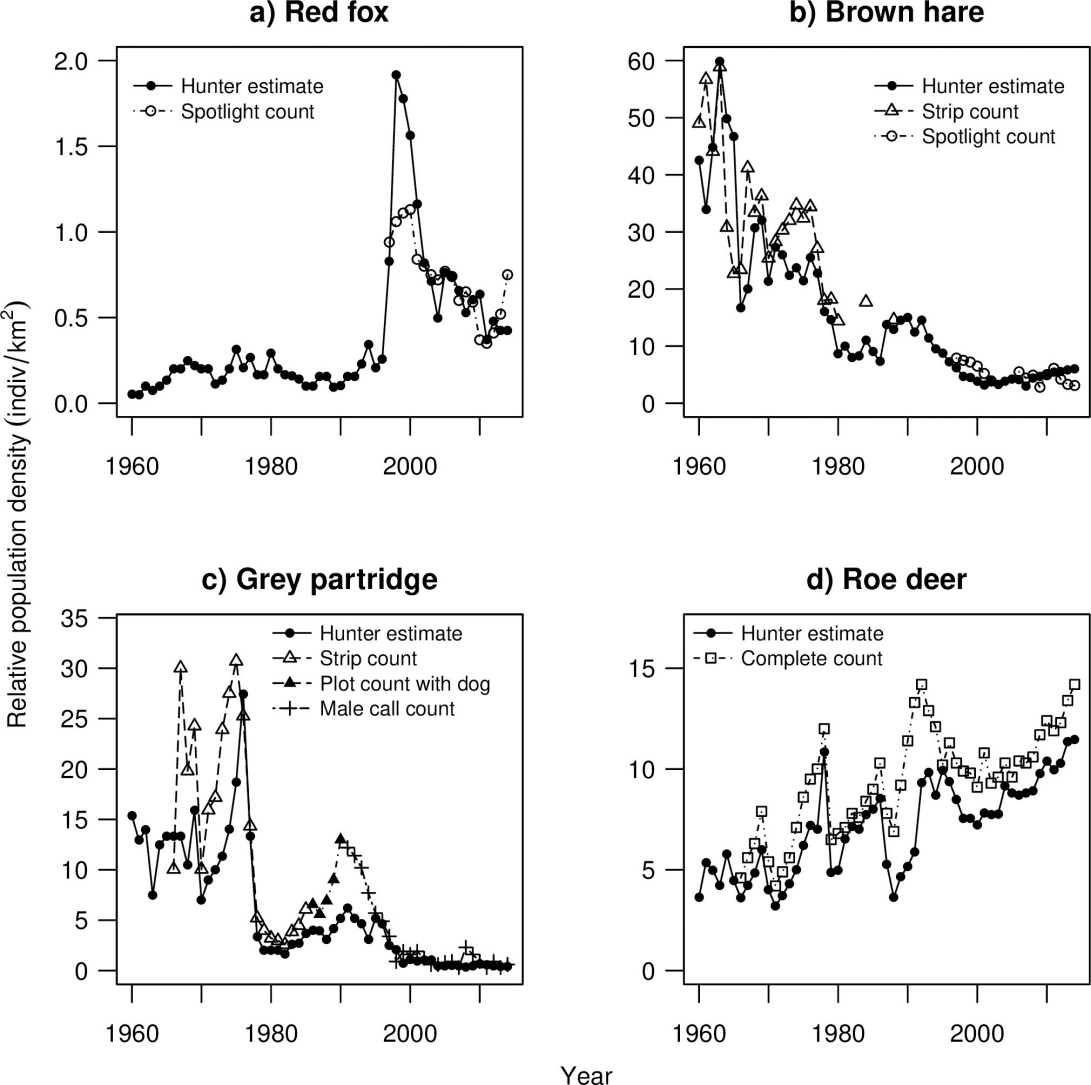
Relative population density based on hunter estimates and estimates derived by other targeted survey methods of a) red fox, b) brown hare, c) grey partridge and d) roe deer from 1960 to 2014 in Czempiń, Poland.

Red fox density was stable at between 0.05–0.3 fox km^-2^ until the late 1990s, when the population underwent a steep increase to 1–2 fox km^-2^, followed by a gradual decline during the 2000s to 0.4–0.8 fox km^-2^ by 2014 (Fig 1A). Brown hare and grey partridge densities decreased steadily from a peak of 60 hares km^-2^ in 1963 to only 2.8–6.1 hares km^-2^, and from a peak of 18.7–30.7 partridges km^-2^ in 1975 down to 0.3–2.3 partridges km^-2^, after 2000 (Fig 1B and 1C). Roe deer density increased steadily from 1960–2014 from 3.6–5.8 to 10.0–14.2 individuals/km^2^ (Fig 1D).

### a) Hunter estimates and estimates from targeted survey methods

**Fig 2:**
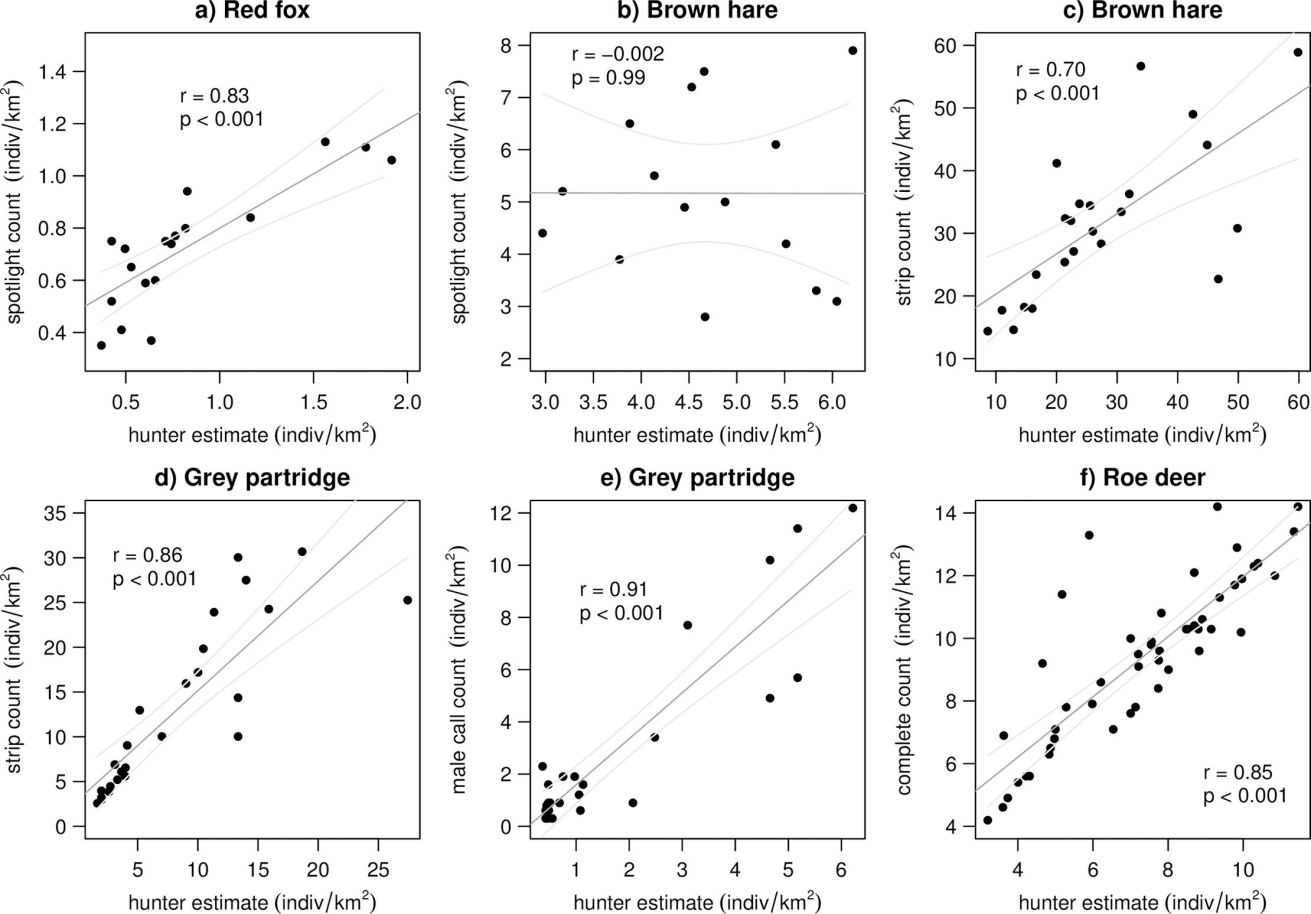
Correlation between hunter estimates of relative population density and estimates of relative density derived by a) spotlight counts from 1997–2014 for red fox, b) spotlight counts from 1997–2002, 2006–2014 for brown hare, c) strip counts from 1960–1980, 1984 and 1988 for brown hare, d) strip or plot counts from 1966–1990 for grey partridge, e) male call counts from 1991–2014 for grey partridge and f) complete counts from 1966–2014 for roe deer. Data from Czempiń, Poland. Line shown is the fit of a linear regression with 95% confidence intervals for illustration purposes only.

For the red fox (*r* = 0.83, p < 0.001, n = 18), but not brown hare (*r* = -0.002, p = 0.99, n = 15), spotlight counts were strongly positively, and not correlated, to hunter estimates, respectively (Fig 2A and 2B). Strip counts were strongly positively correlated to hunter estimates for both the brown hare (*r* = 0.70, p < 0.001, n = 23) and grey partridge (*r* = 0.86, p < 0.001, n = 25) (Fig 2C and 2D). Additionally, partridge male call counts (*r* = 0.91, p < 0.001, n = 24) and roe deer complete counts (*r* = 0.85, p < 0.001, n = 49) were strongly positively correlated to hunter estimates (Fig 2E and 2F).

### b) Realized annual intrinsic population growth rates (*r*_*t*_)

**Fig 3:**
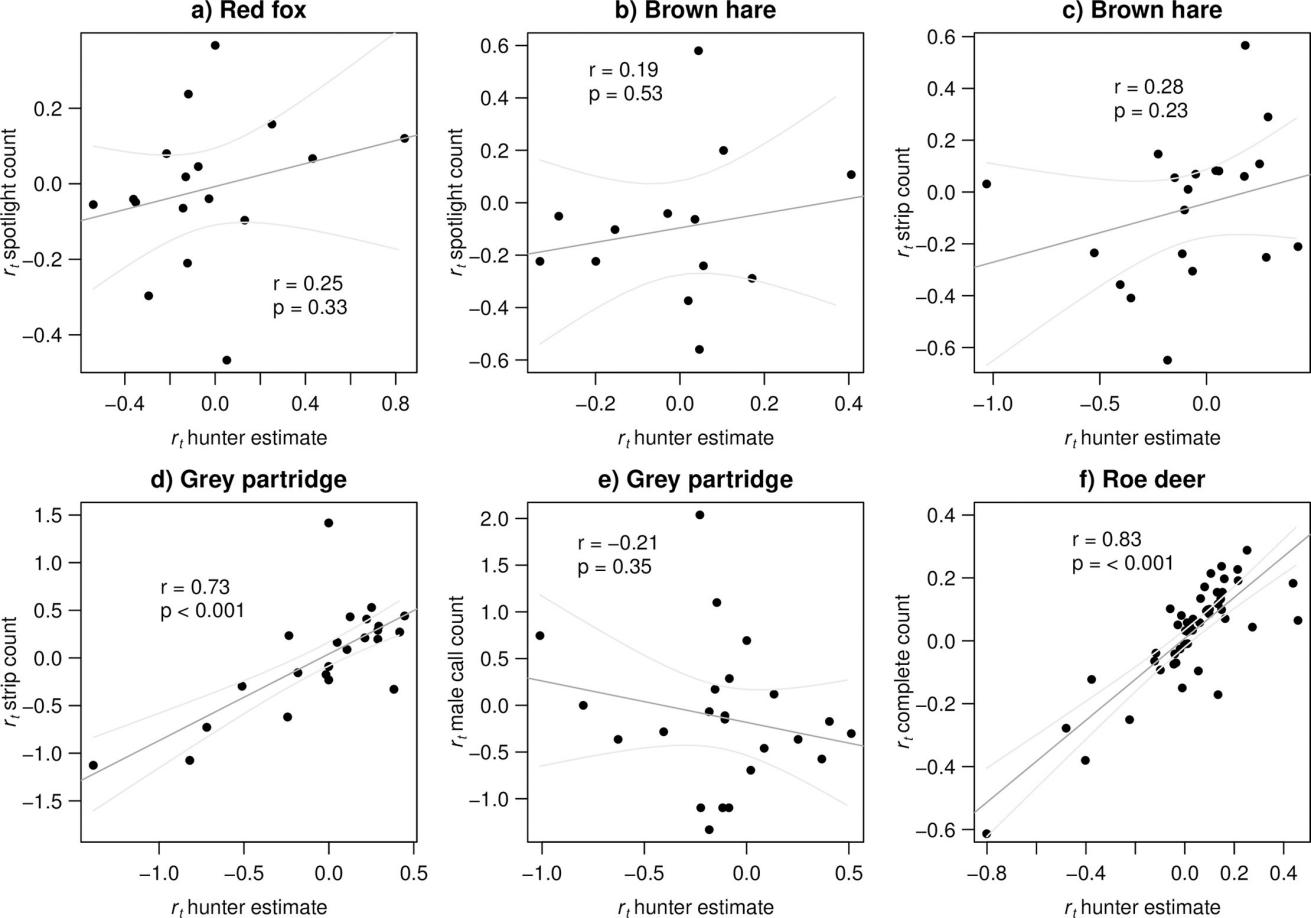
Correlation between realized annual intrinsic growth rate (*r*_*t*_) based on hunter estimates and estimates derived by a) spotlight counts from 1997–2014 for red fox, b) spotlight counts from 1997–2002 and 2006–2014 for brown hare, c) strip counts from 1960–1980 for brown hare, d) strip or plot counts from 1966–1990 for grey partridge, e) male call counts from 1991–2014 for grey partridge and, f) complete counts from 1966–2014 for roe deer in Czempiń, Poland. Line shown is the fit of a linear regression with 95% confidence intervals for illustration purposes only.

Realized annual intrinsic rate of population growth (*r*_*t*_) based on hunter estimates and *r*_*t*_ derived from spotlight counts were not correlated for both red fox (*r* = 0.25, p = 0.33, n = 17) and brown hare (*r* = 0.19, p = 0.53, n = 13) (Fig 3A and 3B). For *r*_*t*_ derived from strip counts and *r*_*t*_ derived from hunter estimates there was also positive but weak correlation for brown hare (*r* = 0.28, p = 0.23, n = 20; Fig 3C), and strongly positive for the grey partridge (r = 0.73, < 0.001, n = 24; (Fig 3D). There was no correlation between *r*_*t*_ derived from male call counts and *r*_*t*_ derived from hunter estimates for partridge (*r* = -0.21, p = 0.35, n = 23; Fig 3E). However, hunter estimate *r*_*t*_ was strongly positively correlated to complete counts *r*_*t*_ for roe deer (*r* = 0.83, p < 0.001, n = 48; Fig 3F).

### c) Mean period intrinsic growth rate (${\bar{r}}_{\mathrm{period}}$)

**Fig 4:**
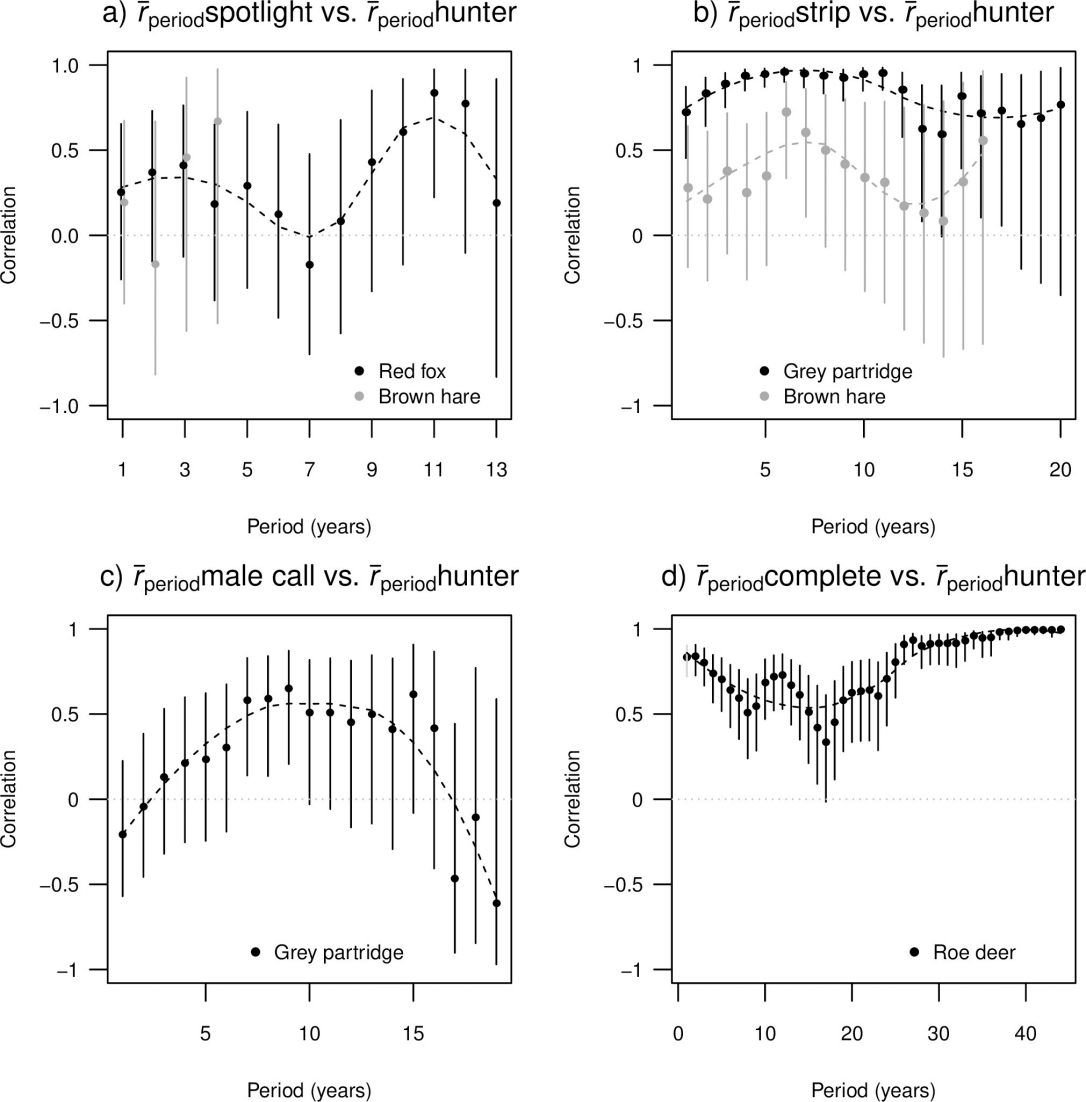
The correlation with 95% confidence intervals between period mean realized intrinsic growth rates (${\bar{r}}_{\mathrm{period}}$) based on hunter estimates and period mean realized intrinsic growth rates based on estimates derived by other targeted survey methods as a function of period length. The dashed line represents a fitted loess smoother to facilitate depicting of apparent trends. Dotted line depicts zero correlation. Period 1 year corresponds to annual growth rates (*r*_*t*_), see Fig 3 for detailed depiction.

The correlation between ${\bar{r}}_{\mathrm{period}}$ derived from spotlight counts and ${\bar{r}}_{\mathrm{period}}$ derived from hunter estimates was strongest when the period length was set to 11 years (*r* = 0.84, p = 0.02, n = 7; Fig 4A). The correlation between ${\bar{r}}_{\mathrm{period}}$ derived from strip counts and ${\bar{r}}_{\mathrm{period}}$ derived from hunter estimates appeared to be strongest when the period was 6 years for both brown hare (*r* = 0.72, p = 0.002, n = 15) and grey partridge (*r* = 0.96, p < 0.001, n = 19; Fig 4B). The period mean growth rate ${\bar{r}}_{\mathrm{period}}$ derived from male call counts was strongly correlated to ${\bar{r}}_{\mathrm{period}}$ based on hunter estimates when the period of 7, 8 and 9 years was considered (*r* = 0.58–0.65, p = 0.016–0.008, n = 15–17; Fig 4C). The correlation between ${\bar{r}}_{\mathrm{period}}$ derived from complete counts and that from hunter estimates was r > 0.3 for all period lengths (Fig 4D). The correlation increased steadily for the periods of 23 years and longer, reaching apparent maximum at a period of 44 years (*r* = 0.99, p < 0.001, n = 5; Fig 4D).

## Discussion

The debate on the robustness of hunter estimates as proxies for game density has yielded mixed conclusions [19,21,22]. Though our study conforms with context dependent legitimacy in approximating game species density based on hunter estimates, it highlights rather neglected, yet important, aspect of hunter estimates in a study of population dynamics: that there is a clear difference in reliability depending on the metric used (i.e., raw estimates, annual growth rates, or period related growth rates). We saw that the raw hunter estimates were mostly strongly positively correlated to estimates from other survey techniques. The only exception was absence of correlation between hunter estimates and spotlight counts of brown hare which may have resulted from data being collected during years of a virtually collapsed local population. Some unease in estimating population density at low population numbers or during steep population declines are also indicated by generally low and more variable correlations between annual growth rates from hunter estimates and male call counts of partridge, strip counts of brown hare, and spotlight counts of red fox.

In larger game, such as roe deer, which are presumably easier to count, we report a strong positive correlation between both raw hunter estimates and raw complete counts along with annual and period mean growth rates. In contrast, for smaller game species, raw strip counts and annual growth rates were more strongly correlated to hunter estimates and annual growth rates in some species (i.e., grey partridge) over others (i.e., brown hares). This variation may be attributed to different antipredator strategies between hares and partridges, given that partridge often exhibit freezing behavior to human disturbance [55], while brown hares may more often choose to run away or hide, increasing the chance they are undetected. Hunters also seem to spot only a small portion of brown hares at low densities and slightly overestimate hares at higher densities as compared to strip counts (Fig 2C). As the hare population has experienced a steep decline over the years, it may be that hunters unconsciously report lower densities in a sort of resignation on hares as a hunted species given their perceptions of hare decline, which is offset during population increases by the temptation to conclude the population is recovering, which may lead to unjustified exaggeration in hunter density estimation.

Compared to the relatively straightforward methodology of visually counting roe deer, hares, and partridges, indirect estimation of fox numbers based on searching for dens demands an additional set of skills from hunters. First, hunters must be perfectly acquainted with the hunting territory to find new, and check existing, dens. Second, determining whether the den is active or not, and identifying residing species (i.e., fox or badger), requires correct interpretation of any sign of activity. Hunter’s estimation of fox numbers based on searching for active dens does not seem to represent a reliable alternative to the estimates obtained from spotlight counts. Even the latter is not without issues. For example, for swift fox (*Vulpes velox*) in the United States, detectability was low using spotlight or visual activity-based surveys [56], while in Australia, red fox detectability was highest using spotlight surveys [57]. A promising way to obtain reasonable estimates of absolute population size in red fox and other elusive small predators without too high additional costs is monitoring by camera trapping [58,59].

Realized annual intrinsic growth rates yielded more variable results across species. It is likely that annual growth rates are sensitive to large yearly fluctuations in density, which was most obvious in the low correlation between *r*_t_ derived from hunter estimates and male call counts of partridge. The former did not seem to sufficiently reflect a peak in density around 1990 (Fig 1C). Yet, period mean realized growth rates (${\bar{r}}_{\mathrm{period}}$) based on hunter estimates showed a strong connection to ${\bar{r}}_{\mathrm{period}}$ based on male call counts for the period 7–9 years. Averaging of the growth rate over given period may be a way to deal with some of the interannual variation. Additionally, determining the proper period length for calculation of ${\bar{r}}_{\mathrm{period}}$ is context dependent and may not always be straightforward. First, it may be severely affected by outliers, especially if those are located towards the beginning or the end of the period. Second it is clearly affected by the species and their population dynamics given that it may be hard to define an informative period length in species with more erratic population dynamics than vice versa. We conclude that, in the latter, the longer the period used for estimation of period mean growth rate, the stronger becomes the correlation between mean growth rates and more similar is the information about population growth conveyed by hunter estimates and complete counts. We also found weak correlation between period mean growth rate derived from hunter estimates and spotlight counts of brown hare and red fox. In these cases, spotlight monitoring was limited only to few years in the 2000s, when the local hare population was at a very low density and fox population was declining steeply rendering density estimation somewhat more difficult. Additionally, although spotlight counts are rather reliable surrogates of true density in the moose [60], the capability of spotlight counts to capture population trends in European rabbit (*Oryctolagus cuniculus*) remains unclear [39,61,62].

Problems in detectability can occur across taxa [63–65] and problems can be exacerbated when detection methods are inefficient and sampling effort is limited [66]. While there is contention on how best to survey species non-invasively, and likely a combination of methods will provide the best results [56], our results show that long-term collections of hunter estimates of population abundance may represent a simple, effective and methodologically sound means to monitor populations when more elaborative and demanding scientific survey methods such as strip counts (i.e., line transects), male call counts, complete counts and platform counts cannot be performed. Given the limited scope of our investigation, and the fact that methods may vary across hunting leases, we promote the idea that there be some sort of standardization in how data are collected to make this information comparable and coherent for use at larger scales. We also acknowledge that none of these types of estimation techniques examined here can tackle the important issues of variability in detection and observer and methodological error [13]. However, we conclude that even under limited understanding of the precision of raw hunter estimates as proxies for true density, the information that may be conveyed from hunter estimates can be largely similar to that conveyed from other survey methods. Indeed, our study was confined to a single hunting territory and may have been affected by turbulent population declines in many small game species that coincided with the study years. Yet, we found consistently positive, and often strong, correlation between raw estimates and/or growth rates from hunters and targeted survey methods among the four game species. These findings bring new light in the discussion about possibilities for both the use of retrospective data, given that these data have been collected for decades in some areas, and future population monitoring efforts. We recommend that game authorities maintain databases of hunter estimates as such long-term data are inherently valuable. Indeed, establishment and maintenance of long-term research programs are typically hard to initiate and maintain, therefore such estimates stand as a good basis to launch for detailed investigations into the factors affecting demographic variability and ecological change over time. Our work supports the view that well-practiced hunter estimates may play a vital role in ecological study, wildlife management, and species conservation.

## Supporting information

S1 Dataset Annual relative densities (individuals km^-2^) of four game species in Czempiń, Poland. Densities were obtained as hunter estimates and estimates from targeted survey methods (i.e., spotlight counts, strip counts, male call counts, and complete counts). These data are plotted in Fig 1.
(CSV)
